# Developmental Changes in Adolescents' Olfactory Performance and Significance of Olfaction

**DOI:** 10.1371/journal.pone.0157560

**Published:** 2016-06-22

**Authors:** Anna Oleszkiewicz, Ute Walliczek-Dworschak, Paula Klötze, Friederike Gerber, Ilona Croy, Thomas Hummel

**Affiliations:** 1 Interdisciplinary Center “Smell & Taste”, Department of Otorhinolaryngology, TU Dresden, Dresden, Germany; 2 Institute of Psychology, University of Wroclaw, Wroclaw, Poland; 3 Department of Otorhinolaryngology, Philipps-University, Marburg, Germany; 4 Department of Psychotherapy and Psychosomatic Medicine, TU Dresden, Dresden, Germany; Instituto Cajal-CSIC, SPAIN

## Abstract

Aim of the current work was to examine developmental changes in adolescents’ olfactory performance and personal significance of olfaction. In the first study olfactory identification abilities of 76 participants (31 males and 45 females aged between 10 and 18 years; *M* = 13.8, *SD* = 2.3) was evaluated with the Sniffin Stick identification test, presented in a cued and in an uncued manner. Verbal fluency was additionally examined for control purpose. In the second study 131 participants (46 males and 85 females aged between 10 and 18 years; (*M* = 14.4, *SD* = 2.2) filled in the importance of olfaction questionnaire. Odor identification abilities increased significantly with age and were significantly higher in girls as compared to boys. These effects were especially pronounced in the uncued task and partly related to verbal fluency. In line, the personal significance of olfaction increased with age and was generally higher among female compared to male participants.

## Introduction

The human sense of smell develops in utero, odors are delivered to the fetus within the amniotic fluid [1]. Already at birth olfaction is functional and smells produce behavioral responses in newborns [2]. For example, newborns show signs of olfactory preferences [3] can distinguish between familiar and unfamiliar odors [4] and are even able to identify their mother by her smell, which is important for feeding and bonding with parents [5]. It is assumed that humans develop olfactory likes or dislikes by evaluative conditioning [6] starting during the prenatal period–although preferences also appear to be partly inherited [7]. The sense of smell helps infants, as well as adults, to gain information about food, environment or people [8]. Studies indicate that body odors influence various regulatory functions related to infants’ motoric functions [9–10] and physiological correlates of distress and arousal [11–13]. Further, infants are able to discriminate the intensity and quality of non-human odors, such as acetic acid, asafoetida, phenylethyl alcohol or anise oil [14,15]. Those abilities seem to constantly develop between the age of 3 and 12 [16]. Studies involving older children have shown that olfactory identification increases further during early and late adolescence and the range of identifiable odorants broadens, partly due to the participants’ verbal abilities to name them [16] and partly due to gaining new experiences [17].

Olfactory identification performance seems to develop until the second decade of life, when it reaches the highest level and it starts to deteriorate after the end of the fifth decade. This might be caused by numerous health-related causes, such as cumulative effect of repeated infections on the olfactory epithelium [18,19].

As yet, most studies concentrated on the developmental aspects of olfactory abilities included infants, older children and adults, however, not many reports included adolescents. Adolescence seems to be the period of human development subjected to dynamic socio-biological changes that directly precedes the peak of olfactory ability. During this period of life, the human body is subjected to the profound biological changes including odor emission and perception [20–23]; as well as changes concerning psychosocial functioning. For this reason adolescence seems to be important period of olfactory development, however the existing literature on olfaction in adolescence is scarce and needs to be supplemented.

Additionally, reports on sex-related differences in olfactory abilities in different age groups, including adolescence, are not conclusive. There are studies suggesting sex-related differences in olfactory abilities (for review see: [24]) and studies presenting contradictory findings [18,25,26]. Some of the tested models managed to explain the sex-related differences through the inclusion of additional variables, such as verbal abilities [16]. To expand the scope of knowledge on olfaction in adolescence, we propose two studies aimed at a) exploration of olfactory identification performance in adolescents and b) analysis of the influence and significance of odors in daily life’s context in this age group.

## Material and Methods

Data were obtained from a total of 207 students (130 females, 77 males) in the age between 10 and 18. The study was performed in accordance to the Declaration of Helsinki on Biomedical Studies Involving Human Subjects. Informed written consent was obtained from caretakers and children participating in Study 1, prior to their inclusion in the study. In Study 2 we only obtained oral consent, as this was an anonymous survey. The study design and consent approach was approved by the University of Dresden Medical Faculty Ethics Review Board (EK 416112014).

The participants constituted the sample of convenience. Statistical analyses was performed by means of SPSS v. 21 (SPSS Inc., Chicago, IL, USA) with *p* < 0.05 set as the level of significance.

## Performance in Odor Identification–Study 1

### Participants

In the study participated 76 students, aged from 10 to 18 years (*M* = 13.8, *SD* = 2.3). Of those, 45 were females between 10–17 years (*M* = 13.6, *SD* = 2.2) and 31 were males between 11–18 years (*M* = 14.1, *SD* = 2.4). To investigate age-related differences in odor identification performance, we divided participants into two age categories: 1) 10–14 years and 2) 15–18 years. This age criterion was made arbitrary and created two equinumerous groups, which was crucial for statistical reasoning. The study was conducted in boarding schools in Dresden, Germany.

### Testing procedure

Six odors of the identification test from the Sniffin’ Sticks test (rose, leather, liquorice, clove, apple, fish) were presented to the participants. The first task was an uncued identification task of the odor, where they were asked to identify the odor without any descriptors given (uncued task). Scores ranged from either 2 points (correct identification) over 1 point (correct odor category, e.g. cloves odor "a “dentistry odor") to 0 points (incorrect identification or far miss, e.g. naming cloves “a spice”). In this task score ranged from 0 to 12 points. The next task was to identify each of the presented odors from a list of four verbal descriptors (cued identification). Here possible scores were 1 point (correct identification) or 0 points (incorrect identification). The range of possible scores was 0–6 points. We used odors from “Sniffin’ Sticks” battery because it is easily understood by participants, it is objective and validated [27]. Further the test is portable. Additionally, taking into account that most of the former studies on olfactory abilities in children used this test, we wanted to deliver comparable results. Results obtained with adult participants suggest, that verbal ability relates to olfactory function [28]. This relationship has been confirmed in self-reported olfactory behaviors [29], but the knowledge on this relationship in adolescents remains limited. To verify whether language development was related to olfactory performance in adolescents an initial letter verbal fluency test was included in this test part. Participants had to list as many words as possible beginning with the letter „b”within 60 seconds [30]. This method of testing language development relies on the assumption that words are organized in memory in first letter order [31]. Thus, performance in this task stems from ease in operating words. Additionally the initial letter verbal test is easy to understand for participants and confirmed to be reliable in studies involving children [30].

### Results

**Fig 1:**
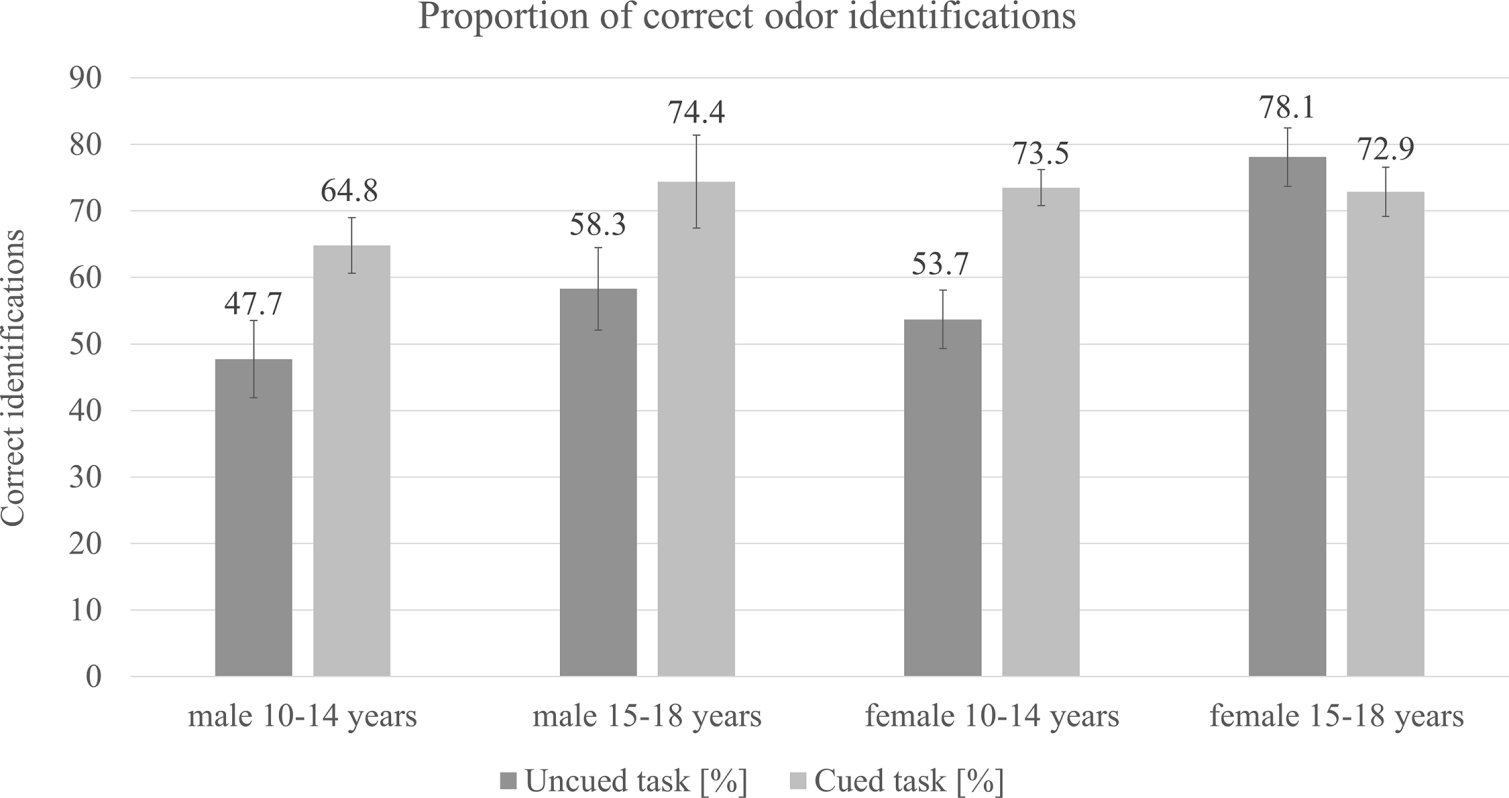
Proportions of correct odor identifications across age and sex groups (in percentages). Error bars illustrate the values of standard error.

To identify the effects of sex, age on odor identification performance and verbal fluency we used multivariate analysis of variance with sex and age as between-subject factors and verbal fluency, cued and uncued identification tasks scores as dependent variables. Analysis revealed a significant main effect of participants’ sex on results in uncued task *F*(1, 72) = 5.7, *p* < .05, *η*2 = .07 indicating higher performance of girls (*M* = 7.5, *SD* = 2.9) in comparison to boys (*M* = 6.3, *SD* = 2.9). No sex-related difference was observed for the cued task (*p* = .39). Similarly, a significant main effect of age was found on uncued identification *F*(1, 72) = 10.5, p < .01, η2 = .13. Pair-wise comparisons indicated higher abilities in free identification among older participants (*M* = 8.3, *SD* = 2.6) than younger ones (*M* = 6.2, *SD* = 2.9). No such difference was observed for the cued task (*p* = .29). There were no significant interaction effects of participants’ sex and age on results in any of the identification tasks (uncued: *p* = .21; cued: *p* = .23) (Fig 1). We found main effect of sex on verbal fluency *F*(1, 72) = 5.3, p < .05, η2 = .07, indicating higher scores among girls (*M* = 11.4, *SD* = 4.9) as compared to boys (*M* = 10.3, *SD* = 2.8). We observed that the verbal fluency increased with age *F*(1, 72) = 25.8, p < .001, η2 = .26. Older children performed significantly better (*M* = 13.7, *SD* = 5) in verbal fluency task than younger children (*M* = 9.3, *SD* = 2.4). For this reason we added verbal fluency as a covariate to the previously tested model. Within the new model, participants’ sex and age had no influence on the results in uncued or cued task and no interaction effect between participants’ sex and age was observed in case of uncued (*p* = .58) or cued identification (*p* = .24) Verbal fluency as a covariate had a significant effect on the uncued task score *F*(1, 71) = 6.9, *p* < .05, *η*^2^ = .09 and no effect on cued task score (*p* = .90). Correlation between uncued task score and verbal fluency was *r* = .47, *p* < .001.

## Personal Significance of Olfaction–Study 2

### Participants

The sample in this study constituted 131 participants, aged from 10 to 18 years (*M* = 14.4, *SD* = 2.2). Of those, 85 were females between 10–17 years (*M* = 14.2, *SD* = 2.1) and 46 were male between 11–18 years (*M* = 14.7, *SD* = 2.3). Participants were divided into two age categories: 10–14 years (N = 82) and 15–18 years (N = 76). This study was conducted in boarding schools in Meissen and Dresden, Germany.

### Testing procedure

The participants filled in a questionnaire assessing the importance of olfaction in their daily life. Items from Croy’s et. al. questionnaire [32] were modified to make them easier to understand for children. The questionnaire consists of the three subscales (each with six items): “Association”, which reflects to emotions, memories and evaluations that are triggered by olfactory experiences (e.g., ‘Certain smells immediately activate lots of memories.’); “Application” referring to the extent to which a person uses his or her sense of smell in everyday life (e.g., ‘‘For tomatoes or pineapples, I find it important how they smell.”) and “Consequence” that reflects the role of olfaction in daily decisions (e.g., ‘‘When I don´t like the smell of a soap, I don´t use it.”) [32]. Each statement in the questionnaire was rated on the scale ranging from 0—*I totally disagree* to 3—*I totally agree* (see: Table 1). Uncompleted items were removed from the data set. Data was transformed in the way, that the higher values indicated higher significance of olfaction. For each subscale summed score was calculated individually.

**Table 1:** Significance of olfaction questionnaire (modified after [32]). Ass–Association; App–Application; Con–Consequence.

*Importance of Olfaction*					
This questionnaire relates to the role your sense of smell plays in your daily life. Please answer all of the questions spontaneously; there are no right or wrong answers.					
Scale		I totally agree	I mostly agree	I mostly disagree	I totally disagree
Ass	I know people, who smell very well.				
App	I do know, how my blankets smell.				
App	I sniff on food before eating it.				
Con	The topic of a history lesson is the old Romanic culture. In an extra lesson you can learn something about the perfumes of this culture. Would you stay in school for this extra lesson?				
Con	When I don´t like the smell of a soap, I don´t use it.				
Ass	When I smell delicious food, I get hungry.				
Con	When I smell smoke on a street, I try to locate the odor.				
Ass	I feel rather quickly disturbed by odours in my environment.				
Ass	Certain smells immediately activate lots of memories.				
App	Before drinking lemonade/tee, I intentionally smell it.				
App	For tomatoes or pineapples, I find it important how they smell.				
Con	If my parents have a nasty smell, I try to stay away.				
Ass	Certain smells make me really happy.				
App	I smell my clothes to judge whether I can still use them or if they need to be washed.				
Con	When there is a nasty smell in a room, I leave the room as soon as possible.				
Ass	Some odours are really interesting to me.				
App	I know, if someone has smoked, because I smell it.				
Con	I love presents which smell well.				

### Results

**Fig 2:**
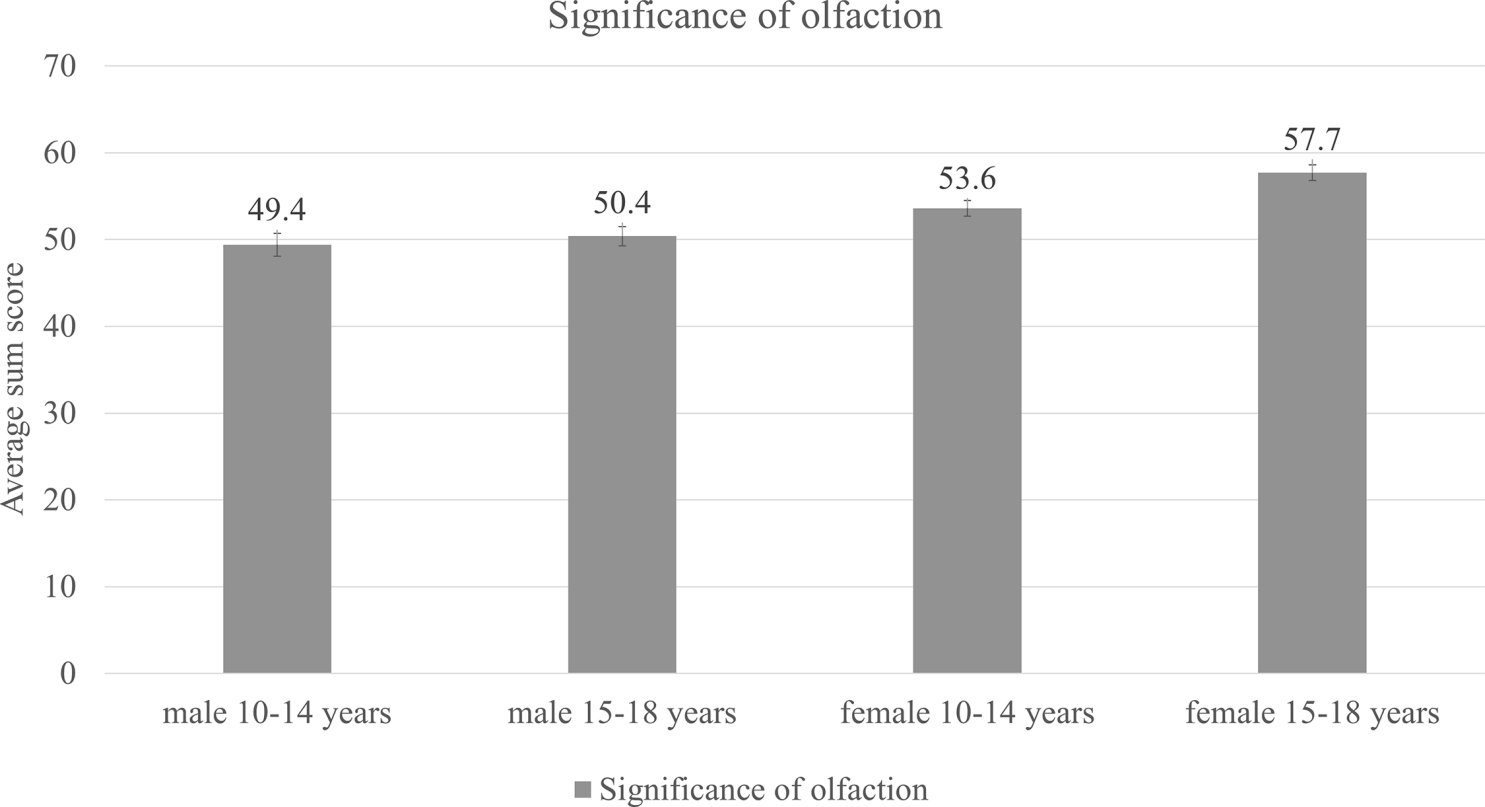
Significance of olfaction across age and sex groups (in units). Error bars illustrate the values of standard error.

In order to examine effects of age and gender on personal significance of olfaction we ran multivariate analysis of variance with age and gender as between-subject factors and results on Association, Application and Consequence subscales of the questionnaires as dependent variables. Analysis revealed significantly higher ratings for girls compared to boys on each of the questionnaire subscales: “Association” *F*(1, 127) = 15.6, *p* < .001, *η*^2^ = .11 “Application” *F*(1, 127) = 10.8, *p* < .002, *η*^2^ = .08 and “Consequence” *F*(1, 127) = 16.6, *p* < .001, *η*^2^ = .12. Significant main effect of age group on “Application” *F*(1, 127) = 6.3, *p* < .05, *η*^2^ = .5 was found (Fig 2).

There were no significant interaction effects between age and sex on any of the three subscales (*p*s > .05). Descriptive statistics for all three subscales can be found in Table 2.

**Table 2:** Descriptive statistics for association, application and consequence (M: mean; SD: standard deviation; in arbitrary units; Min: minimum; Max: maximum).

	Association				Application				Consequence			
Participants	*M*	*SD*	Min	Max	*M*	*SD*	Min	Max	*M*	*SD*	Min	Max
Male	17.2	2.7	9	22	17.3	3.6	6	24	15.5	2.8	10	21
Female	18.9	2.5	12	24	18.9	3.1	11	24	17.7	2.7	10	23
all	18.2	2.7	9	24	18.1	3.4	6	24	16.8	2.9	10	23

## Discussion

Results of the current study indicate that adolescent girls outperform boys in uncued odor identification tasks and that the abilities to name odorants without cues increase with age, which remains in line with former findings [33]. Our results confirmed the significant role of verbal fluency in such changes [16]. In line with these results on odor identification, we found that significance of olfaction in daily life, measured by questionnaire, was higher in girls than in boys and increased with age. This pattern of results indicate, that in adolescence odor identification performance and personal significance of olfaction develop together with cognitive abilities (as verbal fluency suggests) and daily life odor-related experiences gained with age, which are qualitatively different for girls and boys [34].

It seems that olfactory threshold scores stabilize at maximal point in the first decade of life [35], which is earlier in development than maximal identification performance, that follows around the second decade [18]. In line with former studies [24] we noted that in the second decade of life olfactory performance of male and female subjects of similar age was significantly different, in favor for the latter; and that also during adolescence development of olfactory abilities was observable [36], probably due to the older participants’ verbal abilities to name odors. However, the increase in odor identification was observed in uncued task, but not cued task.

Uncued odor identification requires some lexical knowledge and experience in categorizing abstract content [37,38] and hence has higher cognitive load on working memory compared to cued odor identification. It seems possible, that increased olfactory performance is related to higher memory categorization functions allowing to establish association between olfactory stimuli with its verbal descriptor. At the same time, growing mental capacities allow to operate with multiple associations of this kind (as the verbal fluency suggests) [28,39].

With regard to personal significance of olfaction, the young girls generally attached greater importance to olfaction than young boys and different odors affected their daily-life decisions to a greater extent. This finding is in line with adult questionnaire studies using similar [32] or different [40] instruments and a study conducted with 6–10 year old children [29] which indicate that females put more emphasis on olfaction from an early age on. We further observed a significant effect of age on the personal significance of olfaction in daily life context. Emotional association and application of olfaction in daily life increase during the second decade of life (compare: [16]), which is also time of increasing olfactory abilities. We assume the individual significance of olfaction it strongly linked to olfactory performance. It increases with development of olfactory performance and adolescence and it has been shown before that significance of olfaction deteriorates with disease related loss of olfactory functions [41].

In the current study no sex or age-related differences for cued identification task could be detected. In contrast to uncued task (where we observed sex-related differences), cued task does not require the ability to classify odors. Such ability might develop in girls together with their increased interest in odors. Alternatively cued identification task might have been easy for all the participants, regardless of their verbal fluency and odor-related experiences, however we did not observe the ceiling effect in this task (see: Fig 1).

A possible limitation of the current study might stem from the high level of individual differences in participants’ olfactory abilities, that can develop with different speed in puberty. The used age benchmark roughly reflects puberty, although there is high inter-individual and inter-sex variability, that was not controlled in this study.

## Conclusion

Results of this study suggest that odor identification abilities develop with age and accompanying development of verbal fluency. Olfaction is subjectively perceived as more important among girls in comparison to boys.
